# High-efficiency chirality-modulated spoof surface plasmon meta-coupler

**DOI:** 10.1038/s41598-017-01664-w

**Published:** 2017-05-02

**Authors:** Jingwen Duan, Huijie Guo, Shaohua Dong, Tong Cai, Weijie Luo, Zhongzhu Liang, Qiong He, Lei Zhou, Shulin Sun

**Affiliations:** 1 Shanghai Engineering Research Center of Ultra-Precision Optical Manufacturing, Green Photonics and Department of Optical Science and Engineering, Fudan University, Shanghai, 200433 China; 2 State Key Laboratory of Applied Optics, Changchun Institute of Optics, Fine Mechanics and Physics, Chinese Academy of Sciences, Changchun, 130033 China; 3 State Key Laboratory of Surface Physics and Key Laboratory of Micro and Nano Photonic Structures (Ministry of Education), Fudan University, Shanghai, 200433 China; 4 Collaborative Innovation Center of Advanced Microstructures, Nanjing, 210093 China

## Abstract

Efficiently exciting surface plasmon polaritons (SPP) is highly desired in many photonic applications, but most approaches (such as prism and grating couplers) cannot control flexibly their SPP excitation directions. While Pancharatnam-Berry (PB) metasurfaces were recently proposed to achieve direction-controllable SPP excitations, such scheme suffers from low-efficiency issue due to both direct reflections at the coupler surface and the mode mismatch between the coupler and the guiding-out plasmonic structure. In this article, we solve these issues via imposing two criterions to guide design both the metasurface and the plasmonic metal, based on which a direction-controllable SPP excitation with very high efficiency can be realized. As a proof of concept, we designed/fabricated a realistic device working in the microwave regime, and performed both near-field and far-field measurements to demonstrate that it can achieve an spoof SPP conversion efficiency ~78%, much higher than previous devices. Full-wave simulations are in good agreement with experiments, showing that the efficiency can be further pushed to 92% with optimized designs. Our findings can stimulate spoof SPP-related applications, particularly can help enhance the spin-dependent light-matter interactions in low frequency regime.

## Introduction

Surface plasmon polaritons (SPPs), elementary excitations coupled by photons and free-electron oscillations at dielectric/metal interfaces, have attracted intensive attention in the past decades, because of their broad applications in sub-diffraction-limit imaging, biological/chemical sensors, on-chip photonic circuits, and so on^1, 2^. In low-frequency domains where natural SPPs do not exist, spoof SPPs on structured metals can find equal amount of fascinating applications^3–7^. Due to the momentum mismatch, SPPs cannot be directly excited by propagating waves. A grand challenge long-standing in this field is to explore an efficient and controllable way to excite SPPs or spoof SPPs. Conventional optical approaches, such as grating coupler^8, 9^ and prism coupler^6, 10^, typically exhibit low excitation efficiencies^11^ and weak capabilities to control the directions and mode characteristics of the generated SPPs, being inconvenient for integrated-nanophotonics applications.

**Figure 1:**
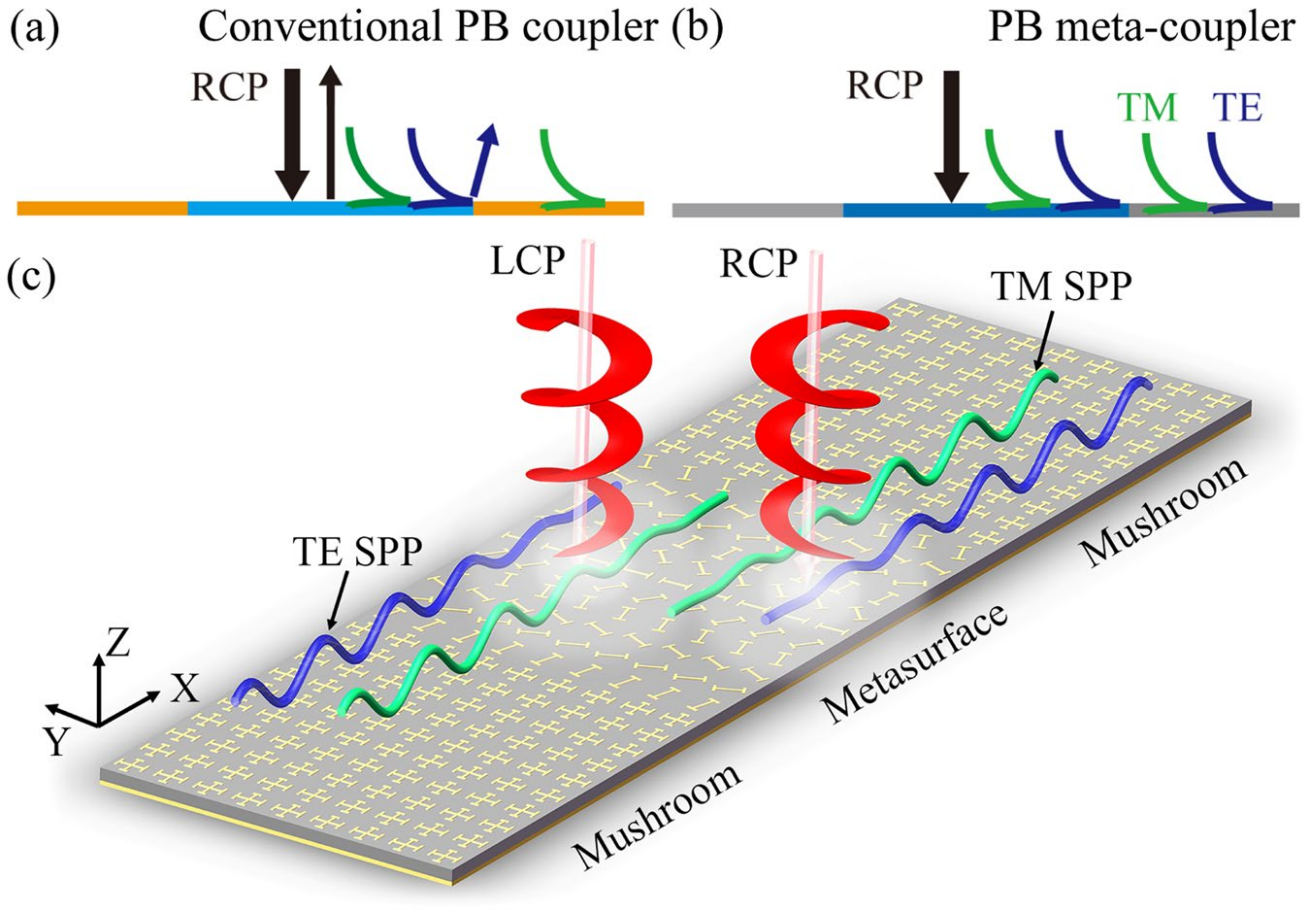
New concept and schematic model of the high-efficiency and direction-controllable SPP meta-coupler. (**a**) Conventional PB coupler consisting of a low-efficiency PB metasurface and plasmonic metals limited by two main issues, i.e., normal reflection and mode mismatch between the driven SWs and SPPs. (**b**) Proposed PB meta-coupler consisting of a high-efficiency PB metasurface and artificial plasmonic metals with previous two issues well solved. Here, the new PB meta-coupler can convert the input CP wave into driven SWs with nearly 100% efficiency, and the artificial metals can guide out both TE and TM polarized SWs to corresponding SPPs with their wavevectors perfectly matching. (**c**) Schematics of the realistically designed PB meta-coupler illuminated by the LCP or RCP waves, producing high-efficiency chirality-controlled SPPs.

Gradient metasurfaces^11–32^, ultrathin metamaterials with tailored transmission/reflection phase distributions, exhibit strong abilities to manipulate the wavefronts of electromagnetic (EM) waves. In particular, employing the phase gradient of the metasurface to compensate the momentum mismatch between free-space radiations and SPPs, various metasurface-based SPP couplers have been recently designed/fabricated in both reflection and transmission geometries and in different frequency regimes^11, 14, 25^. While these meta-couplers indeed show much enhanced SPP excitation efficiencies, the SPP excitation directions are still uncontrolled by external means since the signs of the phase gradients in these metasurfaces are fixed. More recently, researchers demonstrated that geometric-phase metasurfaces (also called Pancharatnam-Berry (PB) metasurfaces) can also couple free-space light with circular polarization (CP) into SPPs, as long as their phase gradients are larger than the free-space wavevectors^33–37^. The most attractive characteristic of such scheme is that the SPP excitation direction can be controlled by the chirality of input CP beam, which dictates the sign of the phase gradient of the PB metasurface. Unfortunately, these PB meta-couplers all exhibit limited SPP conversion efficiencies (typically in the range of 3–15%), due to the following two reasons (see Fig. 1a). First, the specular reflections at such PB couplers are always significant, wasting a considerable portion of input energy (Fig. 1a). Second, while the input CP light contains both transverse-magnetic (TM) and transverse-electric (TE) components, the guiding-out plasmonic structure only supports TM-polarized SPP, and therefore, such mode (or polarization) mismatch again caused non-negligible reflections downgrading the final SPP excitation efficiency (Fig. 1a).

In this article, we establish a new SPP coupling scheme that can realize highly efficient direction-controllable SPP excitations, in which the above-mentioned issues are successfully addressed. In our scheme, the PB metasurface is carefully designed such that it can couple input CP waves into driven surface waves (SWs) with nearly 100% efficiency. Meanwhile, an guiding-out structures metal is also carefully designed such that it can support SPPs with both TE and TM polarizations, which can thus extract efficiently nearly all the energies carried by the input CP light. As a proof of concept, we designed/fabricated a microwave device and then experimentally characterized its functionality. We experimentally demonstrate that the total spoof SPP excitation efficiency achieved by our device reaches 78%, being much higher than previous attempts, while full-wave simulations show that such a value can be even higher (92%) based on further structural optimizations. The robustness, flexibility, and high-efficiency of our spoof SPP excitation scheme can help realize spoof SPP-based applications, particularly related to spin-dependent on-chip light manipulations.

## Results

### New device configuration for high-efficiency PB meta-coupler

Figure 1a schematically illustrates the key issues responsible for the low SPP excitation efficiencies of previous PB meta-couplers — the normal-mode specular reflection at the meta-coupler surface and the mode (or polarization) mismatch between the input CP light and the eigen SPP modes in the plasmonic structure. A careful analysis shows that the SPP excitation process contains two steps — the incident CP light is first converted to a “driven” surface wave (SW) bounded at the coupler surface, which is next coupled into the eigen SPP supported by the guiding-out plasmonic metal^14^. It is thus clear that the above-mentioned two issues can be addressed if we can appropriately design the meta-coupler and the guiding-out plasmonic metal, respectively (Fig. 1b). Specifically, the PB meta-coupler should be designed such that it can convert the incident CP waves into the driven SWs without any normal reflections. Meanwhile, the guiding-out plasmonic metals should also be purposely designed such that they can guide out both TE and TM components of the driven SWs bounded at the meta-coupler. With such appropriately designed structures, a high-efficiency and direction-controllable SPP excitation should in principle be achieved, as shown in Fig. 1c.

**Figure 2:**
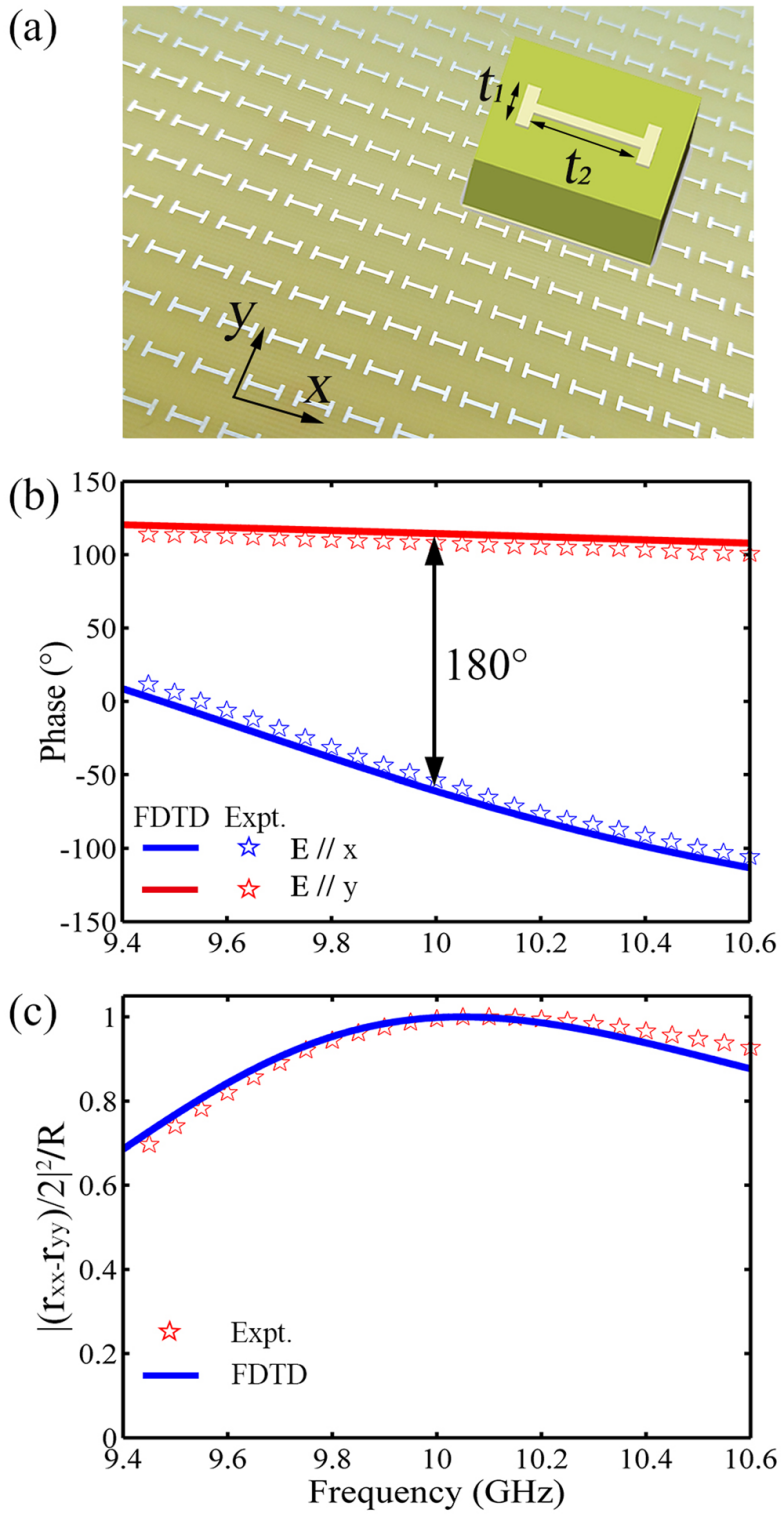
Designs of the high-efficiency PB meta-atom. (**a**) Picture of the PB meta-atoms composed by the symmetrical metallic H structure (sized 7 × 7 mm^2^) array and a flat metal mirror separated by a 2 mm thick dielectric spacer with *t*_1_ = 2.2 mm, *t*_2_ = 4 mm. (**b**) Co-polarization reflection phase of *ϕ*_*x*,*x*_ (blue) and *ϕ*_*y*,*y*_ (red) as a function of frequency for the sample shown in (**a**) illuminated respectively by x and y polarized wave. (**c**) Estimated PW-SW conversion efficiency based on the formula |(*r*_*xx*_ − *r*_*yy*_)/2|^2^/*R* for the sample shown in (**a**) illuminated by CP wave. Here, the results are obtained by both FDTD simulations (solid line) and far-field experiments (open stars). The thickness of the dielectric spacer is 2 mm and the width of the metallic wire is 0.5 mm.

We choose the microwave regime to demonstrate our concept, starting from designing a PB metasurface exhibiting nearly 100% efficiency to convert a CP propagating wave to a driven SW. As discussed in ref. 38, the working efficiency of a PB metasurface depends fundamentally on the Jones matrix of the meta-atom forming the metasurface. In particular, in a totally reflective PB metasurface, the Jones matrix elements of its meta-atom should satisfy the criterion *r*_*xx*_ + *r*_*yy*_ = 0 in order to terminate the normal reflection mode and maximize the efficiency for anomalous reflection mode from the PB metasurface^38^. Such a criterion indicates that the meta-atom should behave as an ideal half-wavelength wave-plate in reflection geometry. We chose a symmetrical anisotropic meta-atom (see Fig. 2a) composed by a metallic H-structure and a flat metal mirror separated by a 2 mm-thick dielectric spacer (*ε*_*r*_ = 4.3 + 0.005*i*), and fix its structural parameters with the help of FDTD simulations. Such tri-layer meta-atom can support magnetic resonances at frequencies dictated by its structural details^39, 40^, thereby it shows strongly frequency-dependent reflection phase spectra for two polarizations. Via carefully optimizing the structural details of the meta-atom (i.e., the arm lengths of the metallic H structure *t*_1_, *t*_2_), we can finally make the system satisfying the desired criterion at a target frequency 10 GHz. To test our predictions, we fabricated a realistic metasurface composed by periodic array of such meta-atoms (see Fig. 2a for the sample picture), and performed microwave experiments to characterize the Jones matrix characteristics of the meta-atoms. Figure 2b shows that, under the illumination of the x-polarized plane wave, the periodic metasurface exhibits a magnetic resonance at about 9.4 GHz leading to dramatic phase modulation within that frequency interval. Meanwhile, the y-polarized magnetic resonance is at a higher frequency (16.5 GHz), so that the y-polarization reflection phase of the metasurface do not vary too much at frequencies around 9.4 GHz (see Supplementary Information for more details). Therefore, at the target frequency 10 GHz, our meta-atom does behave as an ideal half-wavelength wave-plate^20, 38^, as shown in Fig. 2b. Under the illumination of a CP light with a particular helicity, the wave reflected by our meta-atom only contains the helicity-maintained CP component carrying a PB phase, but does not contain the helicity-reversed CP component without the PB phase. Therefore, when we arrange such meta-atoms with orientation angles rotated successively to form a PB metasurface, the normal reflection (without the PB phases) by our metasurface will be completely suppressed leaving only the anomalous reflection survived^38^. Finally, we employed the formula |(*r*_*xx*_ − *r*_*yy*_)/2|^2^/*R* derived in ref. 38 to evaluate the predicted propagating wave (PW)-SW conversion efficiency of a PB metasurface formed by such meta-atoms with orientation angles rotated successively. Figure 2c shows that indeed the predicted PW-SW conversion efficiency approaches 100% at frequencies around 10 GHz, as expected. FDTD simulations are in excellent agreement with experiments.

**Figure 3:**
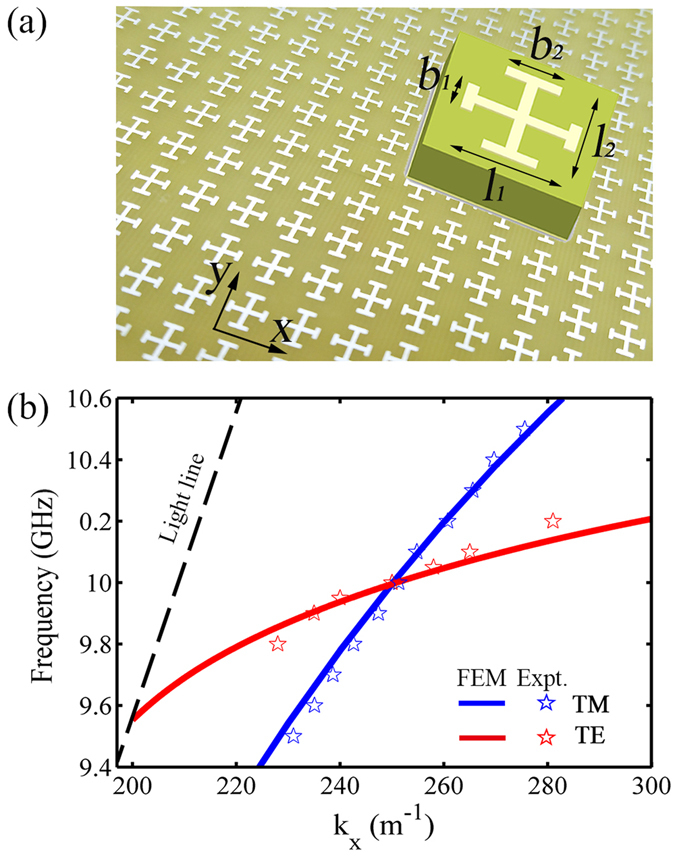
Designs of the artificial plasmonic metal supporting both TE and TM polarized SPPs. (**a**) Sample picture of the plasmonic metal composed by anisotropic mushroom-like structure (sized 7 × 7 mm^2^) array a flat metal mirror separated by a 2 mm thick dielectric spacer with *b*_1_ = 1.5 mm, *b*_2_ = 2.4 mm, *l*_1_ = *l*_2_ = 4 mm. (**b**) FEM simulated (solid line) and measured (open stars) dispersion relations of TE (red) and TM (blue) polarized spoof SPP modes for the mushroom-like plasmonic metal shown in (**a**). The thickness of the dielectric spacer is 2 mm and the width of the metallic wire is 0.5 mm.

We next design an appropriate “plasmonic metal” that can support spoof SPPs with both TE and TM polarizations simultaneously at the target frequency. To match the mode profiles with the coupler and simplify our fabrication, we chose the similar tri-layer structure as the unit cell to design our “plasmonic metal”^14^. As shown in Fig. 3a, the unit cell consists of an anisotropic metallic cross and a metallic plate separated by the same dielectric spacer. It has been shown both theoretically and experimentally that such type of structure (usually called mushroom structure) can support spoof SPPs with TE or TM polarizations under appropriate conditions^39^. The physics can be understood from the effective medium model of such structure, which is a double-layer system consisting of a slab of magnetic material (with effective permeability μ) put on a metal plate^40^. Such magnetic response originates from the anti-parallel electric currents induced on the two metallic layers, under the illumination of the input wave^40^. Obviously, TM-polarized spoof SPPs can be formed in the structure when μ is *positive*, while TE-polarized spoof SPPs can exist only when μ is *negative* (see Supplementary Information for more details). When the upper metallic structure is isotropic (and thus the magnetic slab exhibits an isotropic permeability), spoof SPPs with two polarizations can never co-exist at the same frequency since μ must be either positive or negative. However, the in-plane anisotropy of the upper metallic structure provides us an opportunity to *independently* control the spoof SPP modes with two different polarizations. In fact, for spoof SPPs travelling along the *x* direction, the TM-polarized one only feels the *μ*_*yy*_ component of the $\overleftrightarrow{\mu }$ tensor while the TE-polarized one is sensitive to *μ*_*xx*_. Via fully exploiting the additional freedom provided by the in-plane anisotropy, we carefully optimized the structural parameters (i.e., the arm lengths of the metallic cross structure *l*_1_, *l*_2_, *b*_1_, *b*_2_) of the unit cell and reached our final design based on which a realistic sample was then fabricated (see Fig. 3a for its picture). As depicted in Fig. 3b, FEM simulations show that our “plasmonic metal” can support spoof SPPs with two polarizations within the frequency window. In particular, right at the target frequency 10 GHz, spoof SPP modes with two polarizations exhibit the same eigen wavevector of k_x_ = 251 m^−1^.

### Near-field and far-field verifications of the high-efficiency PB meta-coupler

**Figure 4:**
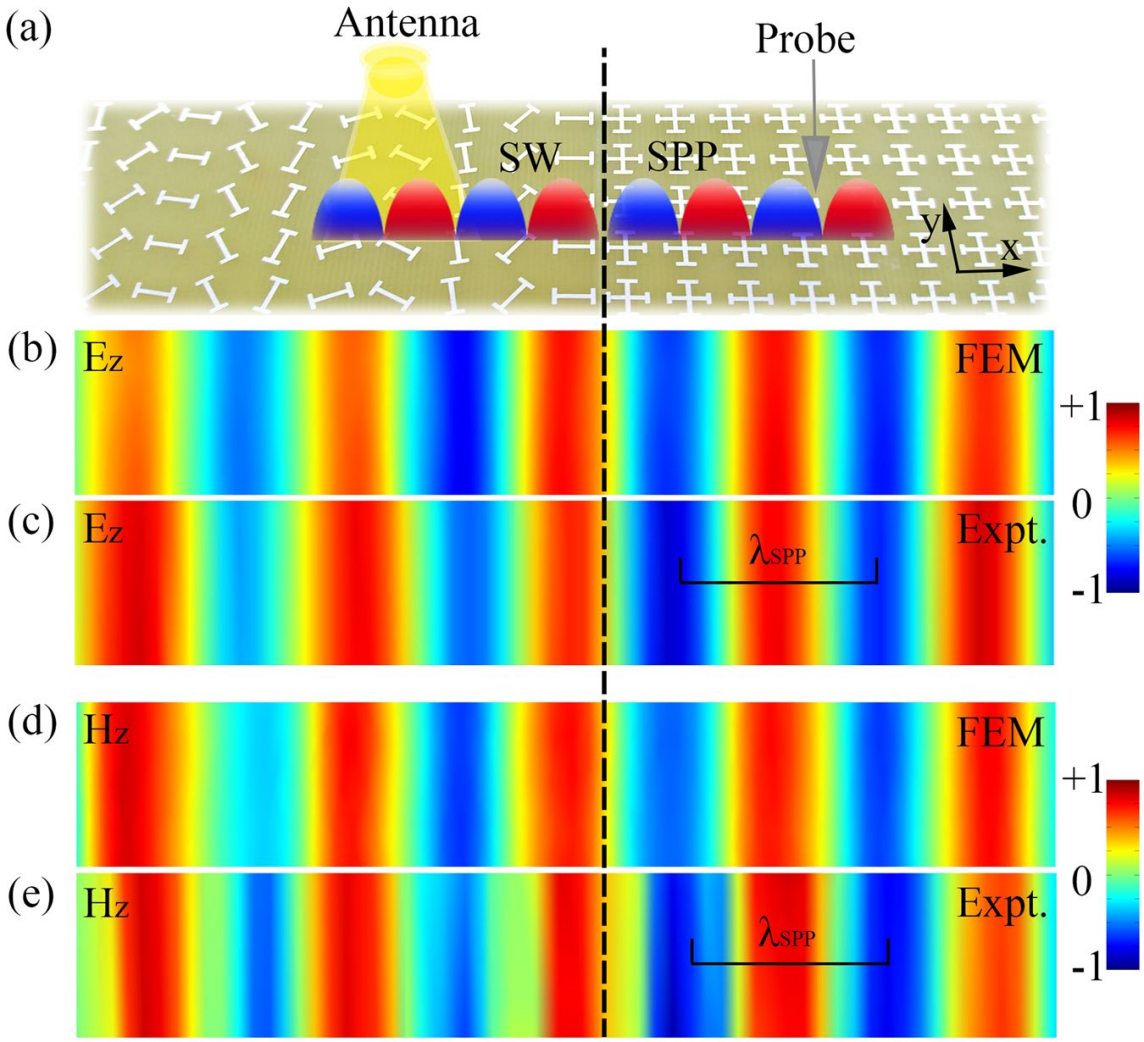
Near-field characterizations of the high-efficiency PB meta-coupler. (**a**) Sample picture and experimental setup to demonstrate the SPP excitation performance of the proposed PB meta-coupler. Using the RCP antenna to normally illuminate the central PB metasurface, we respectively adopted the electric and magnetic probers to measure E_z_ (**c**) and H_z_ (**e**) field distributions (real part) above PB meta-coupler at the frequency 10 GHz, verified well by FEM simulation results (**c**,**e**). Based on the same near-field mapping technique, dispersion relations of TE and TM polarized SPPs on the mushroom (open stars in Fig. 2d) are also retrieved, matching well with simulated results by FEM eigen-mode calculations (see Fig. 3c).

With both the PB meta-coupler and the guiding-out spoof SPP device (i.e., the mushroom structure) designed, we finally build up our high-efficiency chirality-dependent spoof SPP excitation device, with part of sample picture shown in Fig. 4a. In our scheme, a PB metasurface, composed by meta-atoms as designed in Fig. 2 but with local orientation angles rotated successively at a step of *ϕ* = 50.4°, is placed in the center to first couple the incident CP light to the driven SW. Here, each anisotropic meta-atom with a particular rotation angle *ϕ* will totally reflect the input CP light with an additional phase 2*ϕ*, which is called the PB phase^41, 42^. According to the theory presented in ref. 34, such a PB meta-coupler can provide a dispersionless and spin-dependent phase gradient *ξ* = ±2*ϕ*/*a* = ±251 m^−1^, so that it can redirect a normally incident CP light to an EM wave with parallel wavevector *k*_||_ = *ξ*, based on the generalized Snell’s law^12, 14^. At the working frequency 10 GHz, such a parallel wavevector is larger than the free-space wavevector *k*_0_ = 209.6 m^−1^ so that the EM wave “reflected” by the PB meta-coupler is no longer a propagating wave, but rather a SW bounded at the meta-coupler surface (the so called “driven” SW^14^). In addition, the phase gradient of the meta-coupler is purposely designed such that the parallel wavevector of the driven SW precisely matches those of the spoof SPPs on the guiding-out plasmonic structure, thus generating the most efficient coupling for the second process as mentioned in the previous section.

We performed microwave experiments to characterize the performance of the newly proposed SPP coupling scheme. Shining only the central PB meta-coupler part by normally incident right-handed CP (RCP) microwave, we employed near-field scanning technique to measure the local-field distributions on the surfaces of guiding-out plasmonic structures. To unambiguously identify the TM and TE components of the generated spoof SPPs, we purposely adopted two small antennas to respectively probe the E_z_ and H_z_ components of the local fields efficiently. The “electric” antenna is simply a monopole antenna which can efficiently measure the E_z_ field component, while the “magnetic” antenna is purposely designed as a small metallic helix which is very sensitive to the change of H_z_ field component. With these near-field probers at hand, we then placed them on the x-y plane 2 mm away from the device surface, and mapped out the desired near-field distributions of E_z_ and H_z_ components. As shown in Fig. 4b–e, the measured E_z_ and H_z_ field distributions at 10 GHz demonstrated clearly that both TM and TE polarized driven SWs are excited on the PB metasurface and are then efficiently guided out to flow on the mushroom structure as the eigen SPPs with appropriate polarizations. We emphasize that since the normally incident CP plane wave does not exhibit any E_z_ and H_z_ components, such measured near-fields must be due to the excitations of spoof SPPs. Full-wave simulations based on finite-element-method (FEM) are performed on the same system, and the results are in perfect agreement with the experimental results (see Fig. 4b,c). From the measured field patterns, we successfully identified the parallel wavevectors of the spoof SPPs generated on the plasmonic metal at 10 GHz, ${k}_{x}^{TE}\approx 250\,{m}^{-1}$ and ${k}_{x}^{TM}\approx 251\,{m}^{-1}$, which are in perfect agreement with the theoretical values shown in Fig. 3. Encouraged by such agreement, we then employed the same technique to retrieve the whole dispersion relations of spoof SPPs with two polarizations, from the experimental results obtained by varying the frequency of the input CP light. The experimentally retrieved spoof SPP dispersions are shown as open stars in Fig. 3b, which match very well with the FEM-simulated spoof SPP dispersions. Such excellent agreements reinforced our claims that the input CP light has indeed been coupled to the eigen spoof SPPs on the plasmonic structure, aided by our PB meta-coupler.

**Figure 5:**
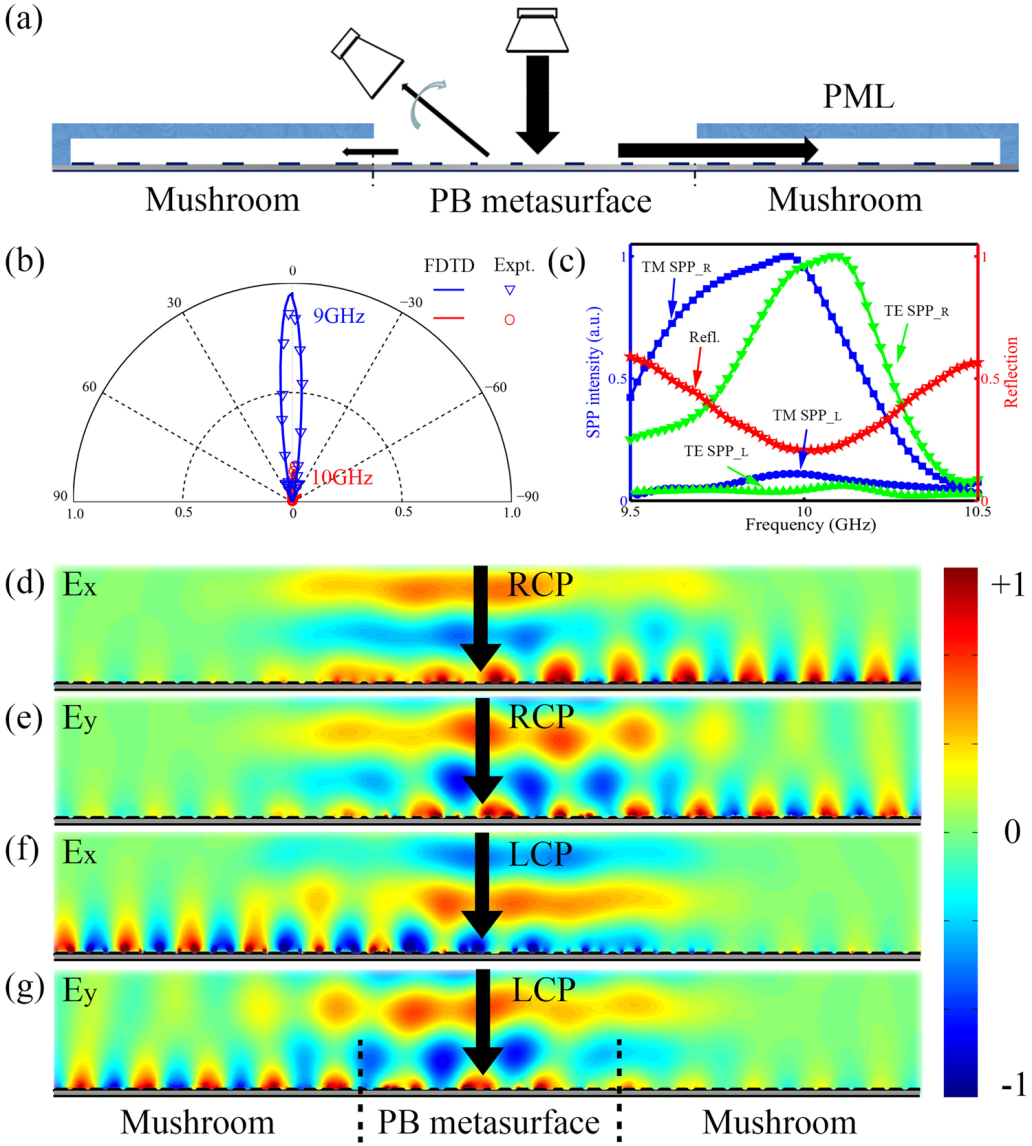
Far-field characterizations of the high-efficiency PB meta-coupler. (**a**) Schematics of the experimental setup for the far-field measurement. Here, we use a right-handed CP antenna to illuminate normally the central PB metasurface and use another CP antenna to collect the scattered field intensity at different reflection angles. (**b**) Measured and simulated normalized angular far-field distributions of scattered waves while the PB meta-coupler is illuminated by input CP wave at 9 GHz and 10 GHz. (**c**) The integrated total reflection R (red, right axis) and the excited SPP intensities (TE SPP_L, TM SPP_L, TE SPP_R, and TM SPP_R) above the left-side and right-side mushrooms (left axis). (**d**–**g**) FEM simulated E_x_ and E_y_ field distributions (real part) inside the SPP meta-coupler shined by a normally incident RCP (**d**,**e**) or LCP (**f**,**g**) Gaussian beam with 2*λ* waist-width at 10 GHz. The SPP excitation efficiency, defined as the ratio between the power carried by the generated directional SPP and that carried by the incident CP Gaussian beam, is about 81% in our simulations.

Having demonstrated that our device can indeed couple a CP light into spoof SPPs, we now quantitatively characterize the efficiency of such a coupling scheme. To achieve this end, we adopted far-field measurements with experimental setup schematically shown in Fig. 5a. When we shine the meta-coupler by a CP wave, three different channels are available to dissipate the input power, which are the total reflection (denoted by R), the absorption (A), and the power carried by the excited SPP (S). According to the conservation law, we can retrieve the spoof SPP excitation efficiency via the formula S = 1 − R − A. To obtain the total reflection R, we adopted a RCP antenna to normally shine the central PB metasurface and used RCP/left-handed CP (LCP) antenna to measure respectively the far-field intensities of waves scattered to angles ranging from −90° to 90°. Figure 5b compares the measured total reflection patterns of our device at two representative frequencies (9 and 10 GHz) normalized against the signal received by a metal plate with the same size under normal illumination. Whereas at frequency 9 GHz (away from working frequency) the device exhibits strong reflections for the input CP light, the reflection becomes significantly reduced at another frequency 10 GHz (central working frequency), implying that most energy carried by the input CP light has been successfully converted into the SPPs. We then integrated the powers carried by scattered waves to all different angles, for our device at different frequencies. However, to quantitatively identify the total reflection R, we need to first calibrate the total input power carried by the input CP light. Therefore, we repeated the measurements with the PB meta-coupler replaced by a metal mirror with the same size, and then performed angle integration to obtain the total power carried by the input CP wave, which is then adopted as our reference. The ratio between the two integrated results is then defined as R, denoting the percentage of incident power scattered by the device. The measured spectrum of R is shown as red stars in Fig. 5c (see more details in Supplementary Information). Right at the working frequency 10 GHz, R becomes significantly reduced (~0.2), indicating nearly all scattered field along different directions are deeply suppressed (consistent with Fig. 5b). To identify where the missing power goes, we further adopted the two probers to measure the spectra of |*E*_*z*_|^2^ and |*H*_*z*_|^2^ at two representative positions above the guiding-out mushroom structures, with the power of incident CP light kept as a constant as frequency varies. Obviously, such measured |*E*_*z*_|^2^ and |*H*_*z*_|^2^ spectra are proportional to the power carried by the excited SPPs with TM and TE polarizations. Figure 5c shows that both |*E*_*z*_|^2^ and |*H*_*z*_|^2^ for SPPs at the right-side mushroom are strongly enhanced at 10 GHz, indicating that the missing power in the R spectrum are coupled to the excited SPPs. Meanwhile, the excited SPPs on the “wrong” side (at the left-side) are negligible, demonstrating clearly the directional SPP excitation property. We further used FEM simulations to estimate that the absorption A of our device is ~2%, based on comparisons with microwave measurements on the Jones matrix characteristics of our samples. With both R and A known, we used the formula S = 1 − R − A to retrieve the experimental value of SPP excitation efficiency, which is about 78% at 10 GHz. As a comparison, we also performed FEM simulations to calculate the field distributions inside structure, as the meta-coupler is shined by a normally incident LCP or RCP Gaussian beam with 2*λ* waist-width (see Fig. 5d–g). The simulated E_x_ and E_y_ field distributions clearly manifest the excitations and propagations of TE and TM polarized spoof SPPs, respectively. With all field information known in FEM simulations, we then integrated the power carried by the generated SPPs with different polarizations and that carried by the input CP wave. The ratio between the two values is just the SPP excitation efficiency, which is found as 81% at 10 GHz with reasonable small loss considered, matching well with the measured value.

Finally, we employed FEM simulations to study the important properties of our scheme and how to further improve the excitation efficiency. We found that the efficiency achieved by our scheme is related to the position on the meta-coupler where the input CP Gaussian beam is shined, as well as the waist-width of the input CP beam. We find that a higher efficiency can be achieved as 92% if we carefully optimize these two factors. More details can be found in Supplementary Information.

## Discussions

In summary, we proposed a new device configuration to achieve highly efficient and direction-controllable SPP excitations, and experimentally demonstrated the concept in the microwave regime. The new device consists of a PB metasurface which can perfectly convert an incident CP wave to a driven SW, and a carefully designed mushroom-like “plasmonic metal” supporting simultaneously TM and TE polarized spoof SPPs with wavevectors matching those of the driven SWs (see more discussions in Supplementary Information). As the result, both the normal reflections at the coupler surface and the decoupling due to the mode mismatch between the driven SWs and the eigen SPPs are significantly suppressed, resulting in highly enhanced SPP excitation efficiencies. We designed/fabricated a realistic device working at 10 GHz, and performed both near-field and far-field experiments to demonstrate that the incident CP light can be coupled into eigen spoof SPPs with an efficiency as high as 78% and a controllable excitation direction via changing the chirality of the incident CP wave. FEM simulations are in good agreements with the measured results, suggesting that the spoof SPP excitation efficiency can be further improved to 92% at an optimized condition. Our findings may not only inspire the realizations of highly efficient and directional-controllable SPP couplers in high-frequency domains (see Supplementary Information), but also help improve the performance of many applications related to high efficiency spin-dependent wave-front manipulations in different frequency domains.

## Methods

### Sample Fabrication

All microwave samples were fabricated using 2 mm-thick printed circuit boards (*ε*_*r*_ = 4.3 + 0.005*i*) with one side covered by a 50 μm-thick continuous copper film and another side covered with the copper patterns etched based on the theoretical designs. For the fabricated meta-coupler, the central metasurface (133 × 399 mm^2^) is surrounded by two mushroom-like structures (175 × 399 mm^2^).

### Far-field measurements

The incident EM waves were generated by a CP horn antenna, and the scattering patterns were measured with co-polarization and cross-polarization CP horn antenna, respectively. Both antennas could be freely moved on a circular track (radius = 1 m) around the sample and were connected to a vector network analyzer (Agilent E8362C PNA). Received signals were normalized against a reference measured when the meta-coupler was replaced by a metal plate of the same size. We have used the absorbing materials to cover the whole mushroom regions to prevent the direct illumination and absorb the generated TE and TM polarized spoof SPPs.

### Near-field experiments

We illuminated the meta-coupler with a CP horn antenna placed at 1 m away from the sample, and then used a electric or magnetic field probe, placed at 2 mm away from the PB meta-coupler and perpendicular to the surface, to measure both the amplitudes and phases of the received local *E*_*z*_ or *H*_*z*_ field on the sample surface. Both antennas were connected to the vector network analyzer.

## Electronic supplementary material

Supplementary Information for High-efficiency chirality-modulated spoof surface plasmon meta-coupler
