# Supplementary material for: High-efficiency chirality-modulated spoof surface plasmon meta-coupler
Source: Sci Rep. 2017 May 2;7:1354. doi: 10.1038/s41598-017-01664-w (PMC5431066; doi:10.1038/s41598-017-01664-w)
Supplement: Supplementary file 1 — Supplementary Information for High-efficiency chirality-modulated spoof surface plasmon meta-coupler [file 41598_2017_1664_MOESM1_ESM.doc]

**Supplementary Information for**

**High-efficiency chirality-modulated spoof surface plasmon meta-coupler**

Jingwen Duan, Huijie Guo, Shaohua Dong, Tong Cai, Weijie Luo,

Zhongzhu Liang, Qiong He, Lei Zhouand Shulin Sun

^1^Shanghai Engineering Research Center of Ultra-Precision Optical Manufacturing, Green Photonics and Department of Optical Science and Engineering, Fudan University, Shanghai 200433, China

^2^State Key Laboratory of Applied Optics ,Changchun Institute of Optics, Fine Mechanics and Physics, Chinese Academy of Sciences, Changchun 130033,China

^3^State Key Laboratory of Surface Physics and Key Laboratory of Micro and Nano Photonic Structures (Ministry of Education), Fudan University, Shanghai 200433, China

^4^Collaborative Innovation Center of Advanced Microstructures, Nanjing 210093, China

1. Details of the designed high-efficiency PB meta-coupler......................................2
2. Additional information of total reflection spectrum in Figure 5c of the main text................................................................................................................................4
3. Beam-position and beam-width dependences of the working efficiency in the proposed PB meta-coupler............................................................................................4
4. Two key issues limiting the performance of the PB meta-couplers.........................6
5. Design of a high-efficiency PB meta-coupler at near-infrared regime.....................7

**A. Details of the designed high-efficiency PB meta-coupler**

In this section, we provide the effective-medium properties of the designed PB meta-atoms and the mushroom-like plasmonic metal. Figure S1a shows the FDTD simulated reflection phase spectra of the designed PB meta-atom (see Figure 2 in the main text) within the frequency domain [5GHz- 18GHz]. It is clear that the PB meta-atom exhibits magnetic resonances at ~9.4GHz and ~16.5GHz for *x* and *y* polarizations, respectively, evidenced by the zero reflection phases at these frequencies (see Figure S1a). The physics of the magnetic resonance can be understood as follows. Under the illumination of an input beam, anti-parallel electric currents will be induced on the top metallic pattern and the bottom metal mirror due to the coupling effect, leading to the formation of a magnetic resonance. Therefore, the meta-atoms can be effectively described by an effective medium model consisting of a 2mm-thick slab of magnetic material (with a dispersive permeability ) put on a flat metal plate^1,2^. On the other hand, the effective permittivity of the capping magnetic material can be chosen as a constant (i.e., ε=1) since the anti-parallel electric currents suppress the electric response of the structure. By fitting with the reflection phase spectrum calculated based on realistic structures, we retrieved the effective permeability of the capping material as $\mu_{xx}=0.5+147.6/(16.5^{2}-f^{2})$, $\mu_{yy}=0.9+63.5/(9.4^{2}-f^{2})$. In the retrieval process, we performed the transfer-matrix-method (TMM) calculations to obtain the reflection phases based on the effective medium model (open stars in Figure S1a). Figure S1b show the retrieved permeability of the PB meta-atoms, exhibiting consistently the magnetic resonance behaviors at 9.4GHz and 16.5GHz (i.e., permeability$\mu\to\infty$).

Figure S2 shows the retrieved effective permeability (i.e., $\mu_{xx},\mu_{yy}$) and the reflection phase spectra of the designed mushroom-like plasmonic metal (see Figure 3 in the main text). The effective permeability is retrieved as $\mu_{xx}=0.5+33/(9.5^{2}-f^{2})$, $\mu_{yy}=0.6+51/(11^{2}-f^{2})$. Specifically, at 10GHz we get that $\mu_{xx}=-2.78$ and$\mu_{yy}=3.03$, which are responsible for the TE-polarized and TM-polarized spoof SPP modes of the designed plasmonic metal, as argued in the main text.

**
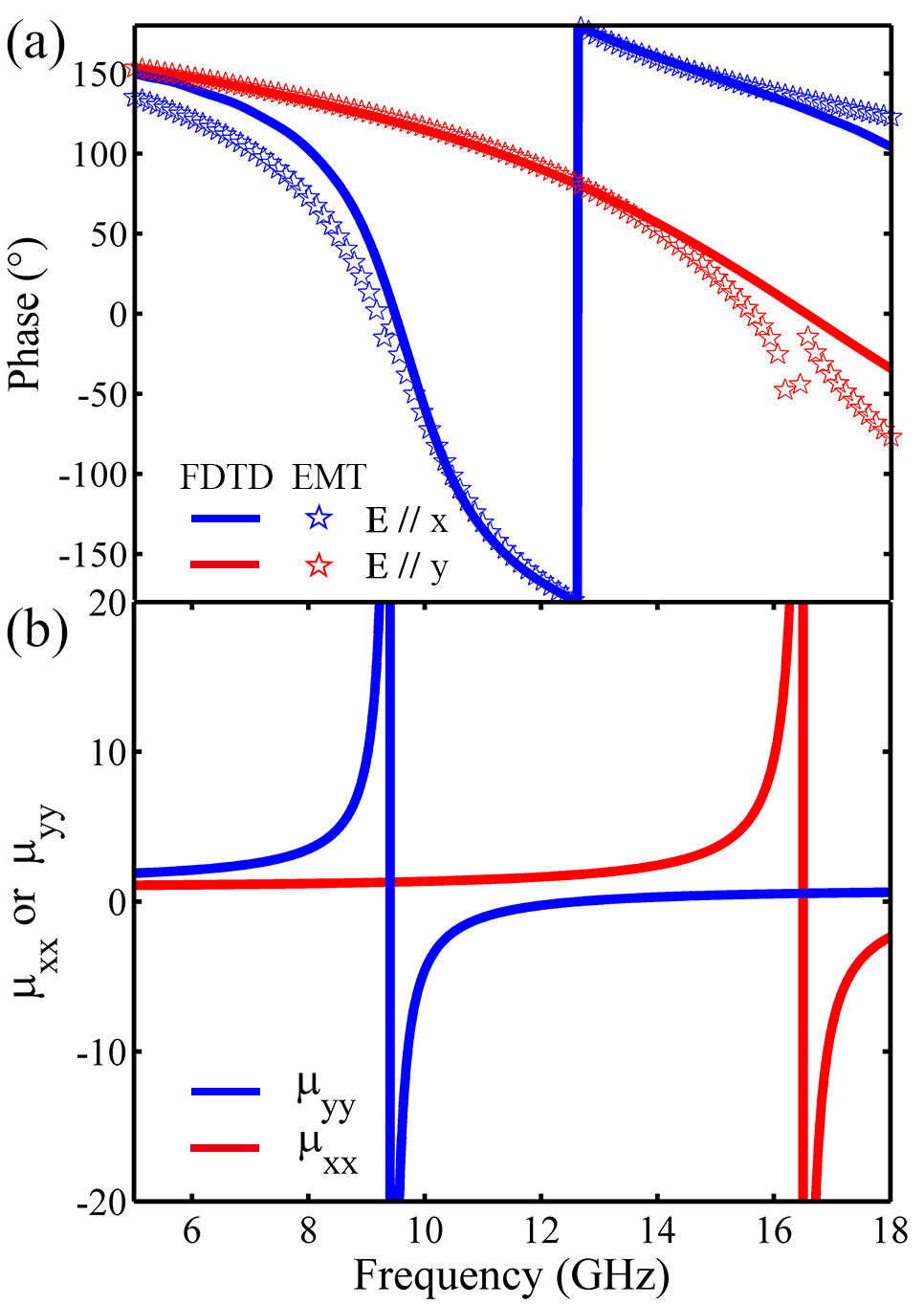
**

**Figure S1**. FDTD simulated/TMM calculated reflection phases (a) and the retrieve permeability (b) of the PB meta-atoms shown in Figure 2 of the main text.

**
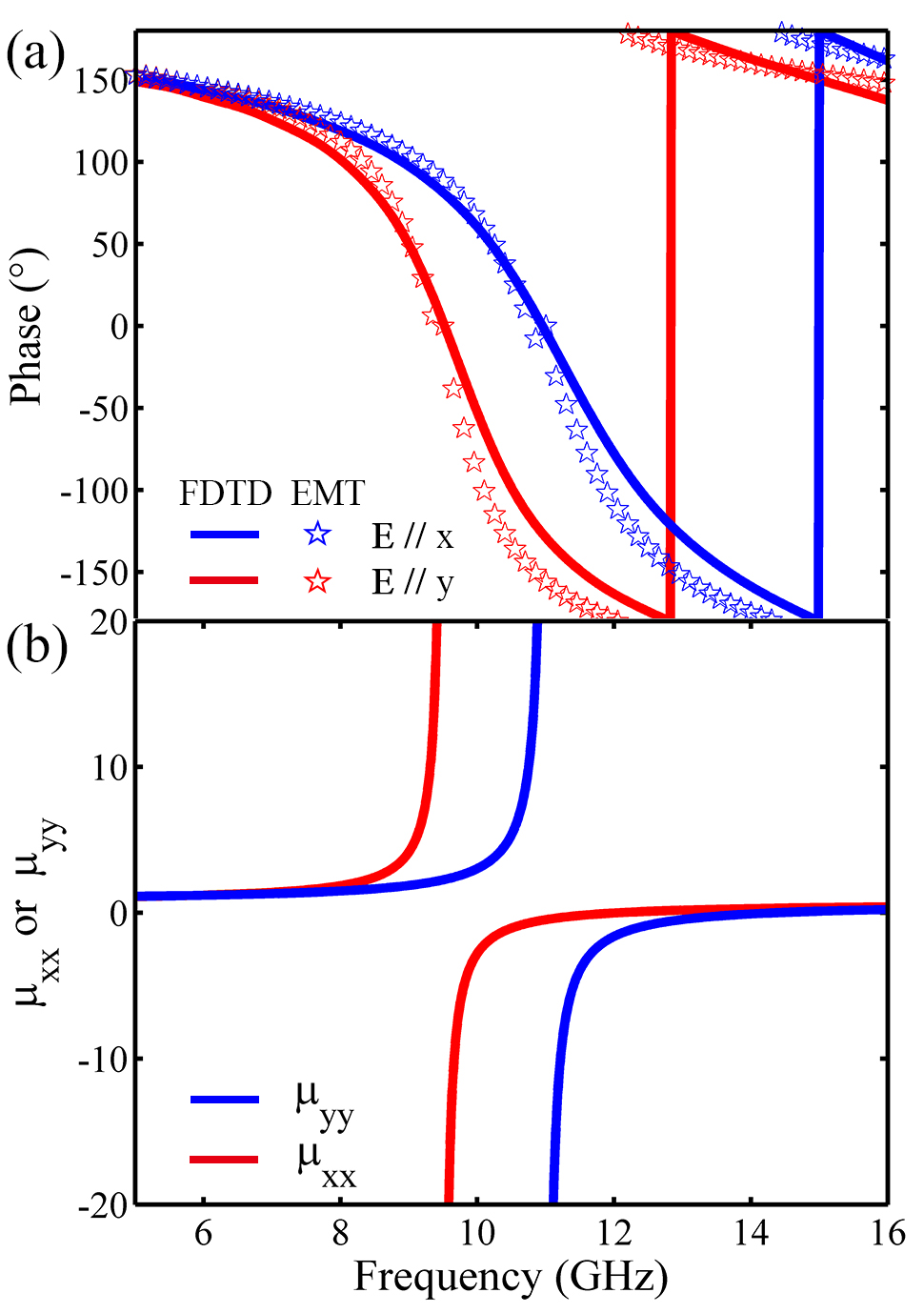
**

**Figure S2**. FDTD simulated/TMM calculated reflection phases (a) and the retrieve permeability (b) of the plasmonic metal structure shown in Figure 3 of the main text.

**B. Additional information of total reflection spectrum in Figure 5c of the main text**

In the far-field measurements, we first adopted RCP antenna to illuminate normally the PB meta-coupler, and used another LCP and RCP antenna to measure respectively the cross-polarization and co-polarization scattering powers at different reflection angles (i.e., from$-90^{\circ}$to $+90^{\circ}$), with the integrated values shown as red circle and green rhombus in Figure S3. Then, we performed the similar measurements with the PB meta-coupler replaced by a flat and same sized metal to obtain the total power of incident RCP beam as the reference. Finally, by summing up the total scattered power of LCP and RCP beams (blue triangle) and dividing it by the total input power of RCP beam (cyan square), we can obtain the normalized total reflection spectrum (red star) in Figure 4c of the main text.


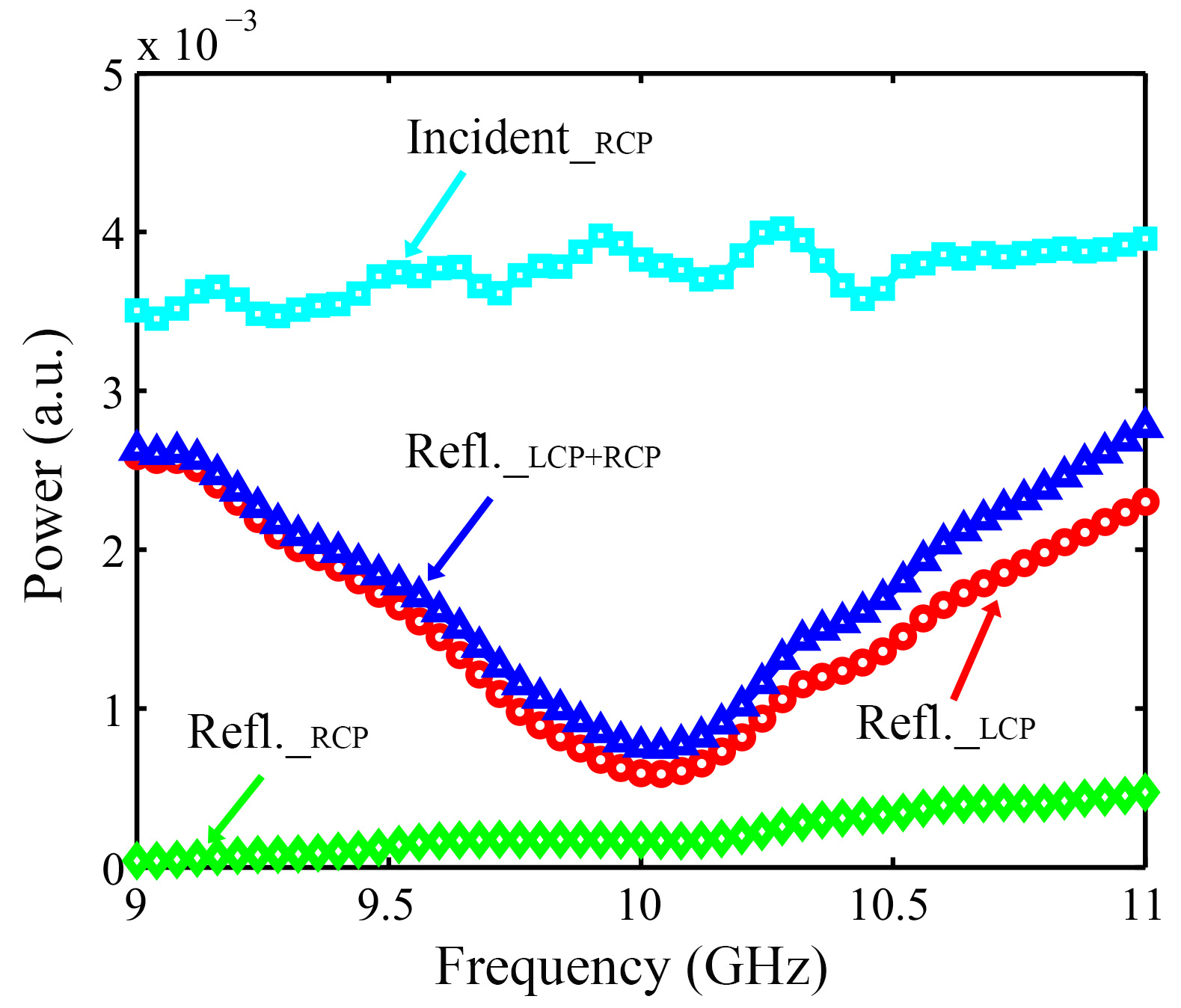


**Figure S3.** The integrated power spectra of the reflected LCP beam (red circle), RCP beam (green rhombus), the total reflection beam (blue triangle), and the input RCP beam for the PB meta-coupler illuminated by the normally incident RCP beam.

**C. Beam-position and beam-width dependences of the working efficiency in the proposed high-efficiency PB meta-coupler**

In this section, we demonstrate numerically that the proposed high-efficiency PB meta-coupler can reach a high efficiency of about 92% at the target frequency 10GHz by optimizing the beam-position and beam-width.

First, we fix the waist-width of incident Gaussian beam as $w=2\lambda$ and calculate the total SPP excitation efficiency as a function of beam-position d (the distance between the beam center and the metasurface center) at 10GHz, as shown in Figure S4. It is obtained by integrating the power flows of the excited spoof SPPs and dividing this value by the integrated power of the Gaussian beam illuminated only on the metasurface part. Obviously, the efficiency is always high and slightly related to parameter d, clarifying the good performance and robustness of our device. Besides, the efficiency increases slowly while the center of Gaussian beam approaches the boundary between the PB metasurface and the mushroom (i.e., d=52.5mm). It implies that the decoupling effect become weaker since the input beam is more easily guided out of the metasurface while the beam center approaches the boundary^3,4^.


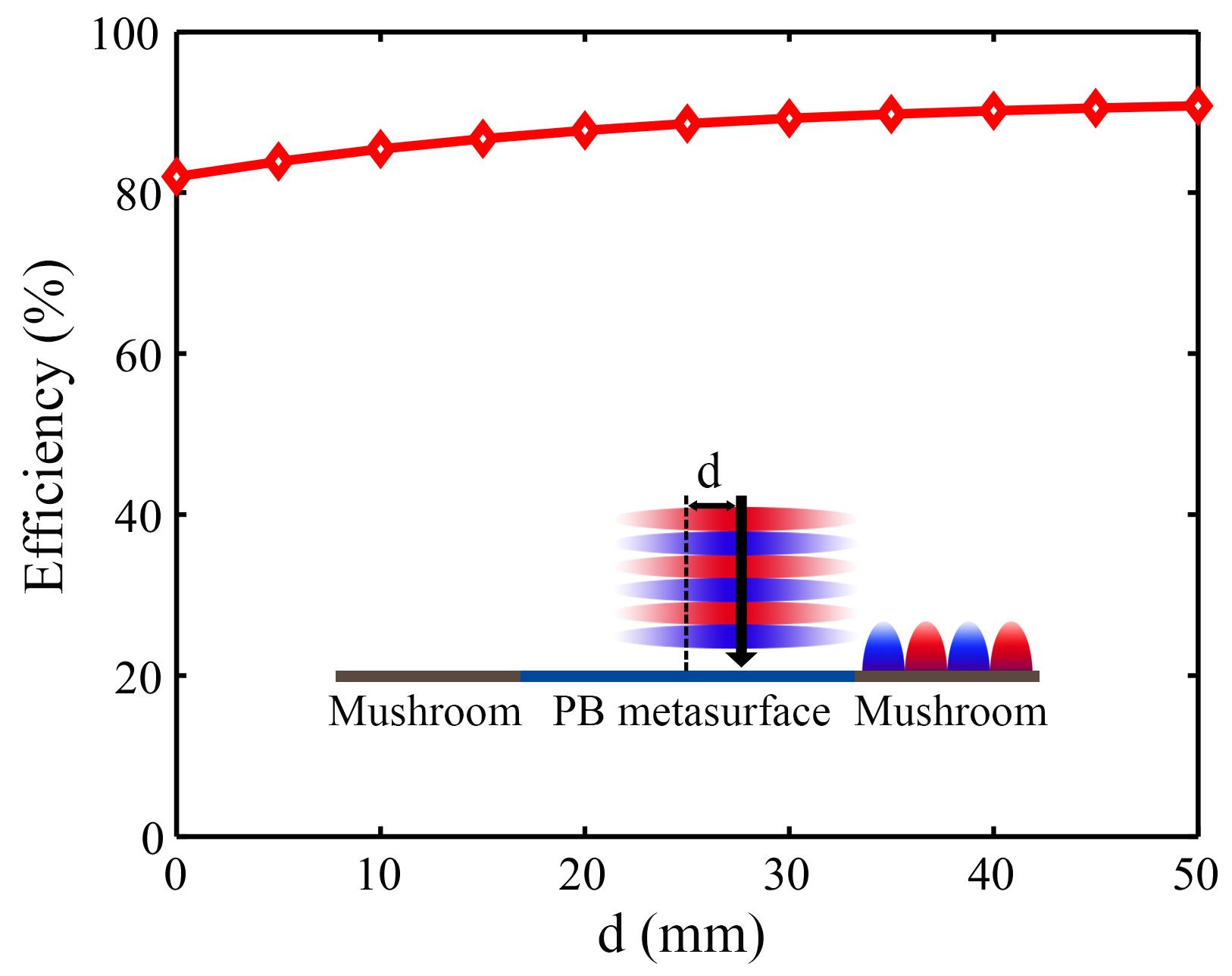


**Figure S4.** FEM simulated SPP excitation efficiency of the PB meta-coupler as a function of distance d. Here, the beam-width of the input Gaussian beam is fixed as $2\lambda$ and the frequency is chosen as 10GHz.

Next, we calculate the SPP excitation efficiency as a function of w, with the beam-position fixed as d=40mm (see Figure S5). It is shown that the SPP excitation efficiency is kept at high value (>70%) with a maximum value of about 92% appearing at w=3.8$\lambda$, demonstrating the good performance of the proposed PB meta-coupler again.


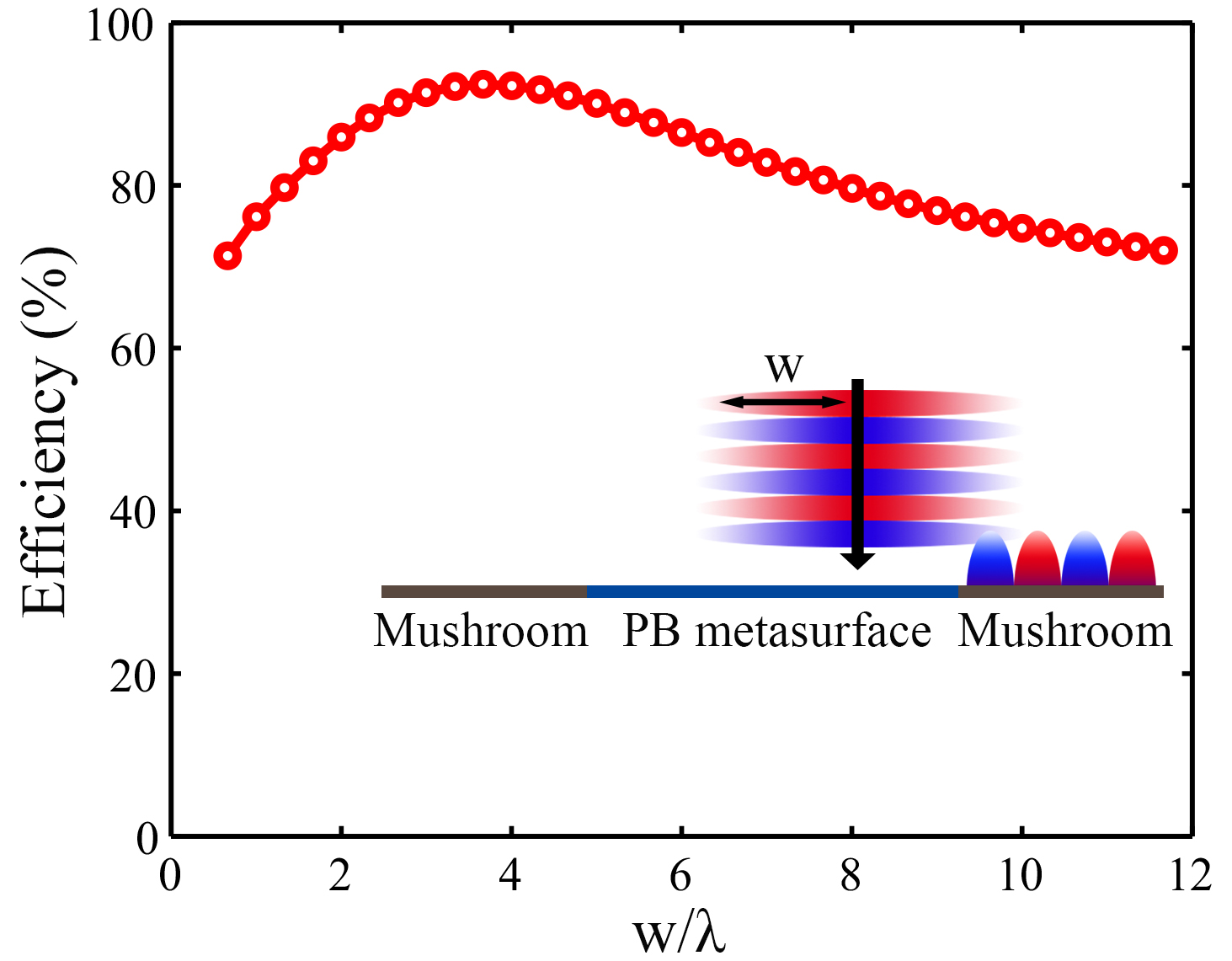


**Figure S5.** FEM simulated SPP excitation efficiency as a function of waist-width w. Here, the position of incident Gaussian beam is fixed as d=40mm, and the working frequency is 10GHz.

**D. Two main issues limiting the performance of the PB meta-couplers**

In this section, based on FEM simulations, we demonstrate that the two main issues, i.e., the normal reflection and mode (polarization) mismatch, do limit the performance of the PB meta-coupler. Here, we compare the performances of three different devices as shown in Figure S6: (a) the proposed PB meta-coupler composed by a high-efficiency PB metasurface and plasmonic metals supporting both TE and TM polarized SPPs (i.e., two main issues both solved); (b) a PB meta-coupler composed by a high-efficiency PB metasurface and plasmonic metals only supporting TM polarized SPP (i.e., only the normal reflection issue solved); (c) a specific PB meta-coupler composed by a low-efficiency PB metasurface and plasmonic metals supporting both TE and TM polarized SPP (i.e., only the mode mismatch issue solved). Obviously, compared to our new meta-coupler (Figure S6a), the meta-couplers with only one issue solved (Figures S6b and S6c) show lower performances, clarifying our claim.


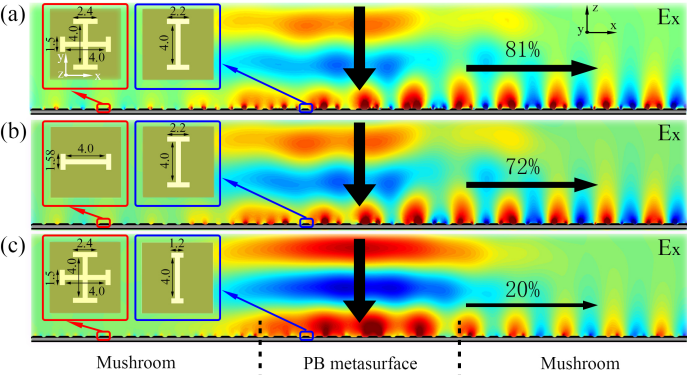


**Figure S6.** Demonstration of the two main issues (i.e., the normal reflection and mode (polarization) mismatch) limiting the performance of the PB meta-couplers. Here, three PB meta-couplers (see insets) show different SPP excitation efficiencies of 81%, 72%, and 20%, illuminated by the same Gaussian beam with the waist-width of w=2$\lambda$at the frequency 10GHz.

**E.** **Design of a high-efficiency PB meta-coupler at near-infrared regime**

In this section, we further extend our idea to near-infrared regime and demonstrate numerically a high-efficiency PB meta-coupler working around the wavelength $\lambda=1.4\mu m$. Figure S7a show the schematics of the designed 400*400nm^2^ PB meta-atom, that are composed by a 350nm-long Au rod and a flat Au mirror separated by a 150nm-thick dielectric spacer ($\varepsilon_{r}=2.35$). Figure S7b shows a specific design of the PB meta-coupler by rotating successfully the meta-atoms with a constant angle step of $\varphi\text{=59}^{\circ}$, supplying a spin-dependent phase gradient of $\xi\text{=}\pm1.146k_{0}$. Here, in order to simplify the fabrication, we use a simple dielectric-on-Au structure to replace the complex mushroom, that can only guide out TM polarized SPP. Even with the polarization mismatch issue unresolved, the SPP excitation efficiency can reach about 60%, going beyond the simulation results in previous works^5,6^. Realizing this idea in the optical frequency is our on-going work.


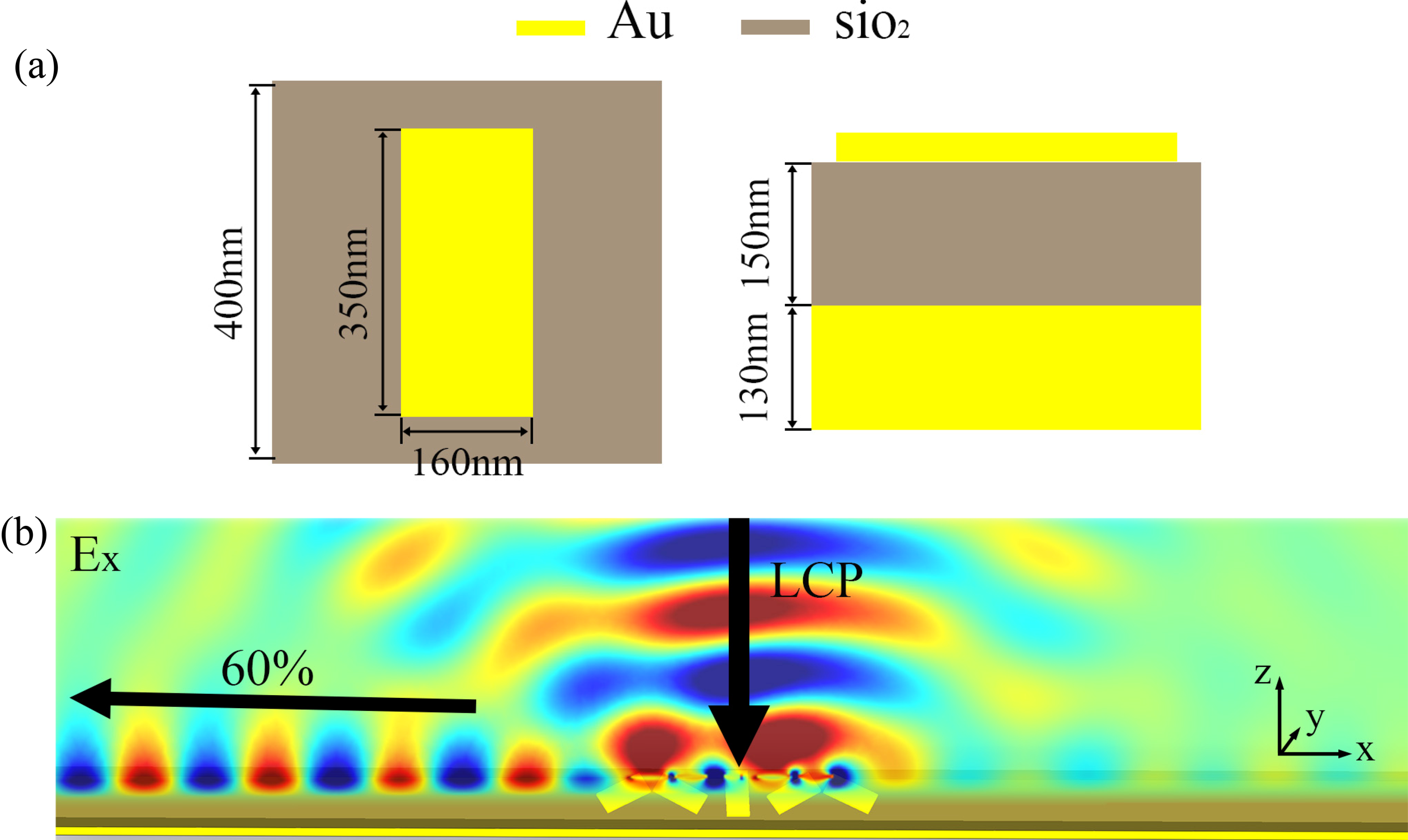


**Figure S7.** (a) Top- and side- views of the designed high-efficiency PB meta-atom**.** (b) FEM simulated E_x_ field distribution inside the PB meta-coupler illuminated by a LCP Gaussian beam at the wavelength $\lambda=1.4\mu m$, showing 60% efficiency to couple TM polarized SPP.

**References**

1. Hao, J., Zhou, L. & Chan, C. T. An effective-medium model for high-impedance surfaces. *Applied Physics A* **87,** 281–284 (2007).

2. Hao, J. *et al.* Manipulating Electromagnetic Wave Polarizations by Anisotropic Metamaterials. *Physical Review Letters* **99,** 63908 (2007).

3. Pors, A., Nielsen, M. G., Bernardin, T., Weeber, J.-C. & Bozhevolnyi, S. I. Efficient unidirectional polarization-controlled excitation of surface plasmon polaritons. *Light: Science & Applications* **3,** e197 (2014).

4. Sun, W., He, Q., Sun, S. & Zhou, L. High-efficiency surface plasmon meta-couplers : concept and microwave-regime realizations. *Light: Science & Applications* **5,** e16003 (2016).

5. Huang, L. *et al.* Helicity dependent directional surface plasmon polariton excitation using a metasurface with interfacial phase discontinuity. *Light: Science & Applications* **2,** e70 (2013).

6. Lin, J. *et al.* Polarization-Controlled Tunable Directional Coupling of Surface Plasmon Polaritons. *Science* **340,** 331–334 (2013).
